# Bombali Virus in *Mops condylurus* Bats, Guinea

**DOI:** 10.3201/eid2509.190581

**Published:** 2019-09

**Authors:** Lyudmila S. Karan, Marat T. Makenov, Mikhail G. Korneev, Noumany Sacko, Sanaba Boumbaly, Sergey A. Yakovlev, Kerfalla Kourouma, Roman B. Bayandin, Anastasiya V. Gladysheva, Andrey V. Shipovalov, Irina A. Yurganova, Yana E. Grigorieva, Marina V. Fedorova, Svetlana A. Scherbakova, Vladimir V. Kutyrev, Alexander P. Agafonov, Renat A. Maksyutov, German A. Shipulin, Viktor V. Maleev, Mamadou Boiro, Vasiliy G. Akimkin, Anna Y. Popova

**Affiliations:** Central Research Institute of Epidemiology, Moscow, Russia (L.S. Karan, M.T. Makenov, Y.A. Grigorieva, M.V. Fedorova, V.V. Maleev, V.G. Akimkin);; Russian Research Anti-Plague Institute, Saratov, Russia (M.G. Korneev, S.A. Yakovlev, S.A. Scherbakova, V.V. Kutyrev);; International Center for Research of Tropical Infections in Guinea, N’Zerekore, Guinea (N. Sacko, S. Boumbaly);; Research Institute of Applied Biology of Guinea, Kindia, Guinea (N. Sacko, S. Boumbaly, K. Kourouma, M. Boiro);; State Research Center of Virology and Biotechnology VECTOR, Kol’tsovo, Russia (R.B. Bayandin, A.V. Gladysheva, A.V. Shipovalov, I.A. Yurganova, A.P. Agafonov, R.A. Maksyutov);; Center of Strategical Planning and Biomedical Health Risks Management, Moscow (G.A. Shipulin);; Federal Service for Surveillance on Consumer Rights Protection and Human Wellbeing, Moscow (A.Y. Popova)

**Keywords:** Bombali virus, Ebola, *Filoviridae*, Angolan free-tailed bats, *Mops condylurus*, *Chaerephon pumilus*, viruses, Guinea, Russia, EBOV, EVD, Liberia

## Abstract

In 2018, a previously unknown Ebola virus, Bombali virus, was discovered in Sierra Leone. We describe detection of Bombali virus in Guinea. We found viral RNA in internal organs of 3 Angolan free-tailed bats (*Mops condylurus*) trapped in the city of N’Zerekore and in a nearby village.

In 2018, a new species of the genus *Ebolavirus* (family *Filoviridae*), Bombali virus (BOMV), was discovered in Sierra Leone (*1*). The virus was detected in oral and rectal swab specimens from 2 free-tailed bat species, *Chaerephon pumilus* (little free-tailed bat) and *Mops condylurus* (Angolan free-tailed bat). Both bat species are widespread in Africa, and their ranges include countries where human Ebola virus disease (EVD) outbreaks have occurred. Forbes et al. (*2*) detected BOMV RNA in mouth swabs and internal parenchymal organs, except kidneys, of *M. condylurus* bats in Kenya in May 2018.

Most known outbreaks of EVD among humans were Zaire Ebola virus, including the large epidemic in West Africa during 2013–2016 (*3*). The reservoir hosts of Ebola virus (EBOV) remain unclear, but bats commonly are suspected. Viral RNA and EBOV antibodies have been detected in a few species of fruit bats (*4*,*5*). The discovery of BOMV supports the hypothesis regarding the role of bats as hosts of EBOVs, but further study is required to determine the bat species involved in viral transmission, prevalence of the virus in bat populations, and geographic distribution of the virus.

We detected BOMV RNA in free-tailed bats in N’Zerekore Prefecture, Guinea. We trapped bats in Guinea and Liberia during 2018–2019 (Table; Appendix) and detected BOMV RNA by reverse transcription PCR in the pools of kidney and lung samples from 2 *M. condylurus* bats captured in Yalenzou village in May 2018 (cycle threshold [C_t_] 17.4 and 19.6) and in a pool of liver and spleen tissues (C_t_ 28.2) of an *M. condylurus* bat from a school in the city of N’Zerekore in March 2019 (Table). Blood, intestine, and brain samples were negative for viral RNA. Sequencing of the 483-bp fragment of the large gene (GenBank accession no. MK543447) demonstrated 99.3% identity with BOMV RNA from Sierra Leone (accession no. NC039345) and 98.3% identity with BOMV RNA from Kenya (accession no. MK340750).

**Table:** Locations where free-tailed bats were trapped and tested for Bombali virus, Guinea and Liberia*

Location	Date	Species, no. tested (no. positive)			
		Total trapped	*Mops condylurus*	*Chaerephon pumilus*	*Chaerephon cf. major*
Yalenzou	2018 May 4	26	26 (2)	0	0
Gbao	2018 May 2	1	0	1	0
Yalenzou	2019 Mar 2	30	30	0	0
Bololowee†	2019 Mar 3	11	11	0	0
N’Zerekore, school	2019 Mar 5	47	27 (1)	0	20
N’Zerekore, house	2019 Mar 6	23	1	0	22
N’Zerekore, gazebo	2019 Mar 7	5	0	0	5
Dar Salam‡	2019 Mar 17	22	14	8	0
Total		165	109 (3)	9	47

*All bats were collected in N’Zerekore Prefecture, Guinea, except as indicated, and were tested by reverse transcription PCR for Bombali virus. †Liberia. ‡Madina Oula Prefecture, Guinea.

Marí Saéz et al. (*5*) suggested that the Angolan free-tailed bat was the most plausible zoonotic source of the EVD epidemic in West Africa. In addition, EBOV nucleotide sequences previously have been found in *Hypsignathus monstrosus*, *Epomops franqueti*, and *Myonycteris torquata* bats in Gabon (*6*). He et al. (*7*) detected filovirus RNA in brown fruit bats (*Rousettus leschenaultii*) in China, and another study showed that 3 distinct groups of unclassified filoviruses are circulating in *Eonycteris spelaea* and *Rousettus* spp. fruit bats in China (*8*). These studies demonstrate that bats are promising targets for identifying emerging filoviruses, and additional Chiroptera species, both insectivorous and fruit bats, should be examined for EBOVs.

EBOV IgG was detected in the human population of Sierra Leone in 2006, 8 years before the EVD outbreak began in that country (*9*). Seroprevalence to EBOVs was also found in the medical staff of hospitals that were not involved in treating EVD-positive patients and in community contacts that worked with villages where EVD was not detected (*10*). The highest seroprevalence to EBOVs was found in the inhabitants of villages with the lowest number of documented EVD cases during the 2013–2016 outbreak in Sierra Leone (*10*). Cross-reactivity or nonspecific binding could be responsible for artifacts of immunoassay. However, other plausible explanations for the presence of antibodies against EBOV among persons with no symptoms of EVD exist, including subclinical EBOV infection in humans and antibody reactions to previously undiscovered, nonpathogenic filoviruses. The newly discovered BOMV could be a causative agent of these types of asymptomatic infections that produce antibodies with cross-reactivity to other EBOVs. Other undiscovered filoviruses also could be circulating in the region. Further surveillance with family-level primers is needed for insectivorous bats, as well as fruit bats and patients with acute infections.

Although BOMV had been detected in the northern part of Sierra Leone (*1*) and in the Taita Hills area of Kenya (*2*), we isolated it from bats in Guinea, far from these sites. Our finding provides additional evidence that BOMV is more widely distributed than previously suspected. Consequently, we advise screening of free-tailed bats for BOMV across their range. The high concentration of BOMV RNA we found in the internal organs of *M. condylurus* bats provides additional confirmation that BOMV could amplify in these bats and that this species is a reservoir host of this virus.

Appendix Additional information on Bombali virus in *Mops condylurus* bats, Guinea.
