# Supplementary material for: Bombali Virus in Mops condylurus Bats, Guinea
Source: Emerg Infect Dis. 2019 Sep;25(9):1774–5. doi: 10.3201/eid2509.190581 (PMC6711222; doi:10.3201/eid2509.190581)
Supplement: Appendix — Additional information on Bombali virus in Mops condylurus bats, Guinea. [file 19-0581-Techapp-s1.pdf]

Article DOI: https://doi.org/10.3201/eid2509.190581

Bombali Virus in *Mops condylurus* Bats, Guinea

Appendix

Methods

Animal Sampling

We trapped bats in the villages of Yalenzou (7°42′29.99″ N, 8°41′20.60″ W) and Gbao (7°32′19.42″ N, 8°46′18.17″ W) in N'Zerekore Prefecture, Guinea in May 2018. In both villages, bats had roosted under the corrugated tin roofs of houses and granaries. We captured 26 free-tailed bats in Yalenzou and 1 bat in Gbao.

In March 2019, we conducted a second trapping session in N'Zerekore Prefecture. We captured 30 free-tailed bats in Yalenzou and 76 free-tailed bats in 3 different sites of N'Zerekore city (a school, a house, and a gazebo in the yard of another house). In addition, we trapped 11 Angolan free-tailed bats in Bololowee village in Liberia (7°41′19.21″ N, 8°40′32.47″ W), and 22 bats in Dar Salam village (9°58′36.88″ N, 12°20′21.08″ W) in Madina Oula Prefecture, Guinea.

We weighed; morphologically described; and measured the length of the head and body, forearm, tail, tibia, hind foot, and ear of trapped bats. Then we genotyped them by sequencing the cytochrome c oxidase subunit I (COI) gene using the following primers: ST-COI-F2 5′-CTCYACYAAWCAYAAAGACATTGGAAC-3′ (*1*) and jgHCO2198 5′-TAIACYTCIGGRTGICCRAARAAYCA-3′ (*2*).

We used cardiac puncture to collect blood into sterile tubes with 0.5 M EDTA. We then euthanized the animals and obtained sections of the brain, liver, spleen, kidney, lung, and intestines through sterile necropsy. In 2018, we collected a blood sample, a pool of lung and kidney tissues, and a pool of liver and spleen tissues from each animal. In 2019, we expanded the sample panel and separately collected the following into sterile tubes: blood; an oral swab; a

rectal swab; and lung, kidney, and intestine samples. Because previous reports did not find viral RNA in the liver or spleen (*3*,*4*), we homogenized these tissues in a single pool for each animal.

We stored blood fractions from N'Zerekore Prefecture at 10°C for 5–7 days, and at –28°C for the next 10 days before studying. We collected tissue samples in RNAlater (ThermoFisher Scientific, https://www.thermofisher.com) and stored them at 6–10°C for 12–15 days before studying. We stored samples from Madina Oula Prefecture at –70°C. We followed standard methods for the safe handling and sampling of small mammals potentially infected with infectious pathogens (*5*).

PCR Array and Sequencing Analyses

We extracted total RNA from blood, brain, and internal organs (lung, liver, spleen, kidney, and intestines) by using RIBO-prep kit (Central Research Institute of Epidemiology, Moscow, Russia, http://www.crie.ru). Then we prepared cDNA by using REVERTA-L (Central Research Institute of Epidemiology, Moscow, Russia). We screened samples for Bombali virus RNA by quantitative reverse transcription PCR by using Filo_UCD_qFor and Filo_UCD_qRev primers, and the Filo_UCD_probe probe sequence (*3*). We amplified cDNA with the Filo-MOD-FWD and FiloL.conR primers, and sequenced it with Filo-MOD-RVS probe (*3*).

References

1. Makenov MT, Karan LS, Shashina NI, Akhmetshina MB, Zhurenkova OB, Kholodilov IS, et al. First detection of tick-borne encephalitis virus in Ixodes ricinus ticks and their rodent hosts in Moscow, Russia. bioRxiv. 2018;480475. http://biorxiv.org/content/early/2018/12/20/480475

2. Geller J, Meyer C, Parker M, Hawk H. Redesign of PCR primers for mitochondrial cytochrome c oxidase subunit I for marine invertebrates and application in all-taxa biotic surveys. Mol Ecol Resour. 2013;13:851–61.

3. Goldstein T, Anthony SJ, Gbakima A, Bird BH, Bangura J, Tremeau-Bravard A, et al. The discovery of Bombali virus adds further support for bats as hosts of ebolaviruses. Nat Microbiol. 2018;3:1084–9.

4. Forbes KM, Webala PW, Jääskeläinen AJ, Abdurahman S, Ogola J, Masika MM, et al. Bombali Ebola virus in *Mops condylurus* bat, Kenya. Emerg Infect Dis. 2019;25.

5. Mills JN, Childs JE, Ksiazek TG, Peters CJ, Velleca WM. Methods for trapping and sampling small mammals for virologic testing. Atlanta: U.S. Centers for Disease Control and Prevention. 1995.
